# Pub1p C-Terminal RRM Domain Interacts with Tif4631p through a Conserved Region Neighbouring the Pab1p Binding Site

**DOI:** 10.1371/journal.pone.0024481

**Published:** 2011-09-08

**Authors:** Clara M. Santiveri, Yasmina Mirassou, Palma Rico-Lastres, Santiago Martínez-Lumbreras, José Manuel Pérez-Cañadillas

**Affiliations:** Department of Biological Physical Chemistry, Instituto de Química-Física “Rocasolano”, CSIC, Madrid, Spain; University of Kent, United Kingdom

## Abstract

Pub1p, a highly abundant poly(A)+ mRNA binding protein in *Saccharomyces cerevisiae*, influences the stability and translational control of many cellular transcripts, particularly under some types of environmental stresses. We have studied the structure, RNA and protein recognition modes of different Pub1p constructs by NMR spectroscopy. The structure of the C-terminal RRM domain (RRM3) shows a non-canonical N-terminal helix that packs against the canonical RRM fold in an original fashion. This structural trait is conserved in Pub1p metazoan homologues, the TIA-1 family, defining a new class of RRM-type domains that we propose to name TRRM (TIA-1 C-terminal domain-like RRM). Pub1p TRRM and the N-terminal RRM1-RRM2 tandem bind RNA with high selectivity for U-rich sequences, with TRRM showing additional preference for UA-rich ones. RNA-mediated chemical shift changes map to β-sheet and protein loops in the three RRMs. Additionally, NMR titration and biochemical *in vitro* cross-linking experiments determined that Pub1p TRRM interacts specifically with the N-terminal region (1–402) of yeast eIF4G1 (Tif4631p), very likely through the conserved Box1, a short sequence motif neighbouring the Pab1p binding site in Tif4631p. The interaction involves conserved residues of Pub1p TRRM, which define a protein interface that mirrors the Pab1p-Tif4631p binding mode. Neither protein nor RNA recognition involves the novel N-terminal helix, whose functional role remains unclear. By integrating these new results with the current knowledge about Pub1p, we proposed different mechanisms of Pub1p recruitment to the mRNPs and Pub1p-mediated mRNA stabilization in which the Pub1p/Tif4631p interaction would play an important role.

## Introduction

RNA binding proteins (RBP) play essential regulatory roles during different stages of mRNA metabolism. Poly(A)-binding protein 1 (Pab1p) [1], [2] and poly(U)-binding protein 1 (Pub1p) [3], [4] are among the most abundant mRNA binding proteins in *Saccharomyces cerevisiae*. These two proteins recognise RNA sequences with high affinity and specificity by using modular architectures that contain several RNA binding domains, a strategy commonly used by RBPs. Pab1p and Pub1p contain respectively, four and three RNA-recognition motifs (RRMs), a 80–150 amino acids domain very abundant in higher eukaryotes [5], [6], [7]. RRMs can also function as protein-protein recognition domains, as has been already shown in several complex structures (reviewed in [7]). The second RRM of Pab1p interacts with Tif4631p and Tif4632p, the eIF4G1 and eIF4G2 homologues in yeast [8], [9], promoting the formation of a “closed-loop” structure for the mRNA, which stimulates translation initiation by facilitating mRNA recruitment to pre-initiation complexes [10], [11], [12]. The “closed-loop” mRNP is recruited to the 43S pre-initiation complex (43S PIC) to form the 48S PIC and represents a crucial checkpoint in the regulation of translation initiation (recently reviewed in [13]). Proteins that bind to eIF4E or Pab1p can lead to destabilization of the “closed-loop” mRNP causing translation initiation arrest [13], [14].

Pub1p biological function antagonizes the Pab1p one. This protein is not associated with polysomes, but rather seems to form part of the translationally inactive mRNAs pool [3]. Pub1p is homologous to mammalian HuR and TIA-1/TIAR proteins, which modulate mRNA stability by blocking different degradation pathways. Pub1p also causes changes in mRNA turnover, majorly stabilization [15], which is regulated by Pub1p recognition of RNA sequences in the 3′- and 5′-UTRs. For example, Pub1p recognises stabilizer elements (STE) in the 5′-UTR protecting GCN4 and YAP1 mRNAs against the NMD pathway [16], but can also bind ARE-sequences in the 3-UTR preventing deadenylation-meditated mRNAs degradation [17]. Moreover, Pub1p and TIA-1 proteins play also an important role in translational repression [18], [19]. Protein expression arrest is an universal mechanism of response to stress (recently reviewed in [20]) and is frequently regulated by cellular signalling pathways that lead to post-translational modification of proteins involved in translation initiation: eIF2α and 4E-BPs among them [13]. Microscopically, stress-induced translational arrest causes the accumulation of most of the mRNAs into cytoplasmic RNA granular structures such as stress granules (SG) and processing bodies (PB) in both yeast and mammalian cells [21], [22], [23], [24], [25], [26]. In general, cytoplasmic mRNPs are in a dynamic equilibrium between actively translated forms (i.e. polysomes) or arrested states (i.e. SG, PB and other RNA granules). Different stressors cause inhibition of translation initiation at different stages, altering this balance and causing the accumulation of different types of RNA stress granules. Formation of stress granules has been reported in *Saccharomyces cerevisiae* upon glucose starvation (also known as EGP-bodies for eIF4E,G and Pab1p) [27], [28], [29], [30], robust heat shock [31], arsenite [30], sodium azide [32] and high ethanol levels [33]. Pub1p is required for the formation of the glucose deprivation RNA granules (i.e. EGP-bodies) [25], [27], [29]. In addition to Pub1p, this type of RNPs contain Pab1p, Tif4631p, Tif4632p, Tif45p, Pbp1p, Ngr1p, Ygr250c, Gbp2p and Nrp1p proteins, but lack components of the 43S pre-initiation complex (including the 40S ribosomal subunit and eIF3), an important difference with the mammalian SG [22], [23]. Heat shock stress granules have been recently reported in yeast [31] and are compositionally more similar to mammalian SG. However their mechanism of assemble is independent of eIF2α phosphorylation, in contrast to the mammalian ones. Like in the EGP-bodies the heat shock stress granules contain Pub1p. The influence of this protein in the nucleation of SG is likely due to the presence of a prion-like C-terminal domain, which in the case of TIA-1 has been probed to be necessary for in the SG formation [34], [35], [36]. Nam8p and Ngr1p, the other *S. cerevisiae* TIA-1 homologues [4], [37], also contain this prion-like domain. Nam8p plays a role in mRNA splicing [38] that resembles that of TIA-1 [37], [39], [40], [41], [42] while Ngr1p is the yeast homolog of TIAR [29].

Here we have studied the structure and protein-RNA and protein-protein recognition properties of different regions of Pub1p by NMR. The protein contains two RRMs at the N-terminus whose X-ray structure has been recently reported [43]. We have assigned the NMR spectra of this region and determined that it binds prefentially poly(U) sequences with both RRMs participating in the RNA recognition. We have also determined the solution structure of the C-terminal RRM domain, which adopts an atypical fold with a novel N-terminal helix that is chiefly stabilized by the interactions with a key tryptophan residue. Remarkably, these unique features seem to be exclusive of the Pub1p/TIA-1 protein family. This single RRM displays low micromolar affinity for U-rich and UA-rich sequences with a slight preference for the latter ones and the recognition mediated by the β-sheet and the loops. Furthermore we have discovered that the Pub1p C-terminal domain recognises specifically the N-terminal region (residues 1–402) of Tif4631p. We have characterized this interaction by NMR spectroscopy, following changes in signals of both Pub1p and Tif4631p, and by *in vitro* protein-protein cross-linking experiments with glutaraldehyde. NMR chemical shift mapping experiments identify a novel protein-protein recognition surface, which involves residues conserved in Pub1p/TIA-1 proteins. Comparative cross-linking experiments on a Pub1p mutant confirm the interaction and validate the NMR-identified interface. Additional NMR experiments that monitor Tif4631p (1–402) signals allowed us to assign the Pub1p binding site to a conserved peptide box (Box1) that contains the single Trp residue (Trp 95) of the molecule. The new structural information is integrated with previous biochemical data to propose possible models of Pub1p-mediated mRNA stabilization and translational control

## Results

### NMR characterization of Pub1p constructs

*Saccharomyces cerevisiae* Pub1p is a 453 residue protein that contains three RRM domains. We cloned three fragments of this protein: Pub1p R13 containing RRM1, RRM2 and RRM3 domains (residues 32–414), Pub1p R12 including RRM1 and RRM2 domains (residues 32–242) and Pub1p R3 that contains RRM3 (residues 315–414). The constructs were expressed as thioredoxin A fusions (N-terminal) to enhance protein expression and solubility. Nevertheless, only Pub1p R12 and Pub1p R3 expressed as soluble proteins and remained so after tag removal. Pub1p R3 is less soluble (300 µM) than the Pup1p R12, and becomes even less soluble at pH below 7.0, maybe due to contributions of histidine side chains titrating in this pH range. Pub1p R13 construct expressed into inclusion bodies, perhaps due to the asparagine/methionine-rich region in the RRM2-RRM3 linker. Other aggregation-prone sequences (like the N- and C-terminal poly-glutamine regions) were not included in the designs. Remarkably, prion-like regions are required for mammalian stress granule assembly in TIA-1 [34], [36], the metazoan homolog of Pub1p.

**Figure 1:**
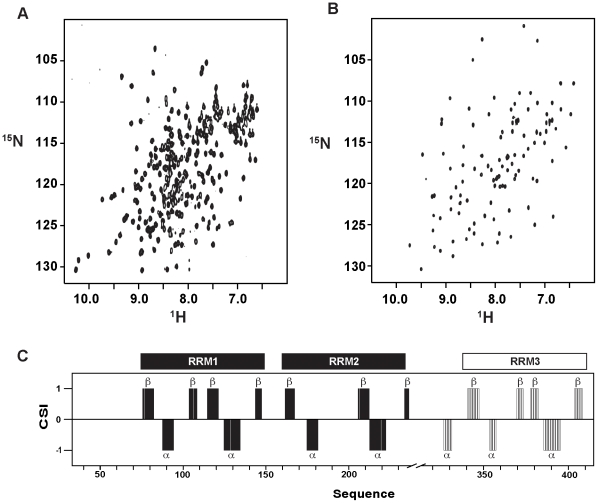
NMR spectra of Pub1p constructs and secondary structure prediction. (**A**) ^1^H-^15^N HSQC spectrum of the Pub1p R12 construct (0.5 mM DTT, 25 mM NaCl, 25 mM potassium phosphate buffer, pH 6.5) at 25°C. (**B**) ^1^H-^15^N HSQC spectrum of the Pub1p R3 domain (0.5 mM DTT, 25 mM NaCl, 25 mM potassium phosphate buffer, pH 7.0) at 25°C. (**C**) Consensus chemical-shift index [44] calculated for both Pub1p R12 (black) and Pub1p R3 (white) proteins using the CSI program (http://www.bionmr.ualberta.ca/bds/software/csi/latest/csi.html). The predicted α-helical and β-sheet regions are marked in the histogram with α and β, respectively.

Pub1p R12 and Pub1p R3 constructs are monomeric and well-folded according to the appearance of their ^1^H-^15^N HSQC spectra (Figure 1), proton amide T_2_ relaxation measurements, gel filtration chromatography and, in the case of Pub1p R3, sedimentation equilibrium ultracentrifugation experiments (data not shown). The chemical shift index CSI [44] provides evidences about the secondary structure contents of these proteins (Figure 1C). Crosspeaks corresponding to linker residues between RRM1 and RRM2 (149–157) are missing in the ^1^H-^15^N HQSC spectrum, evidencing that this segment might be conformationally flexible in the ms to µs time scale. Interestingly, the structure of a Pub1p construct similar to ours (residues 75–240) shows that this linker participates in the formation of a crystallographic swapped dimer [43]. Our studies found no evidence about the presence of such dimer in solution, in agreement with the analysis of gel filtration data performed by the authors of crystal structure. Nevertheless, the line broadening effect might be explained with the hypothesis of a chemical equilibrium between a major monomeric and a minor dimeric forms occurring in an intermediate time scale (ms to µs). Finally, the first N-terminal 41 residues (32–72) of Pub1p R12 (not included in the X-ray construct) are conformationally disordered and highly mobile as derived from the chemical shifts and sharp line shapes.

For Pub1p R3, the most remarkable feature extracted from the ^13^C chemical shift analysis is the prediction of a non-canonical helical region right before the first strand of RRM3. Hence we decided to pursue a full structural determination of this protein domain.

### Pub1p R3 fold defines a novel RRM structural class

**Figure 2:**
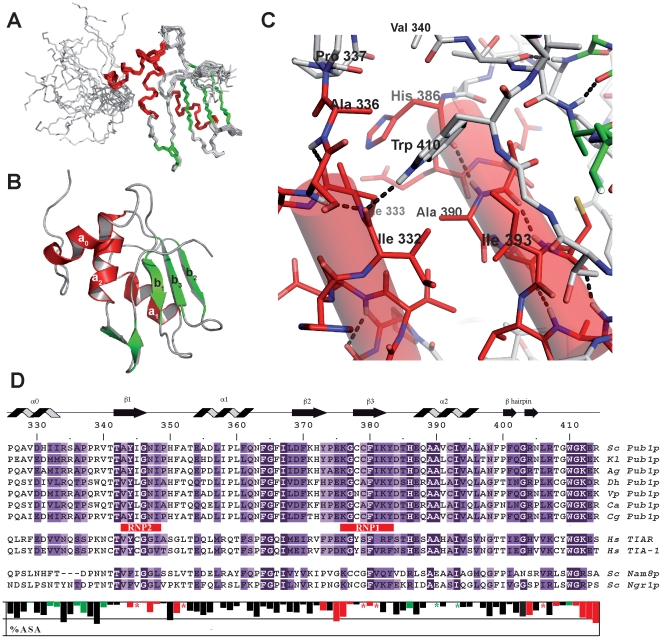
The NMR structure of Pub1p R3 shows novel features. (**A**) Backbone atom superposition of the Pub1p R3 20 NMR calculated structures with the lowest target function (PDB:2LA4). (**B**) Ribbon plot of one representative NMR structure of Pub1p R3 with secondary structure elements numbered and depicted in green (β-strands) and red (α-helices). (**C**) Detailed view of the structure around α_0_ and α_2_ helices. Trp 410 and other residues that form the hydrophobic core are labeled and hydrogen bonds are represented as dashed donor-to-acceptor lines. (**D**) Sequence alignment of fungal Pub1p (Sc: *Saccharomyces cerevisiae*, Kl: *Kluyveromyces lactis*, Ag: *Ashbya gossypii*, Dh: *Debaryomyces hansenii*, Vp: *Vanderwaltozyma polyspora*, Ca: *Candida albicans* and Cg: *Candida glabrata*), human TIA-1/TIAR and *Saccharomyces cerevisiae* Nam8p and Ngr1p proteins. Secondary structure elements in Pub1p R3 structure are indicated on the top of the panel and the sequences have been highlighted in different shades of purple according to the level of conservation found across Pub1p/TIA-1 proteins. Typical RNP1 and RNP2 motifs found in RRM domains are also indicated. A histogram showing the percentage of accessible surface area (ASA) per residue is plotted on the bottom of the figure. Bars corresponding to residues involved in (UA)_7_ binding (Δδ^av^≥0.1 ppm. See text and Figure 4) are highlighted in red and those taking part of the hydrophobic core of α_0_ helix are depicted in green.

Pub1p R3 NMR structure (PDB: 2LA4) was calculated from 1968 NOE-derived distance restraints and 129 angular restraints obtained from the analysis of selected ^13^C chemical shifts (Table 1). Excluding the first ten residues of the construct and the last two, which can be regarded as disordered, the structure (Figure 2) is of high quality with very low RMSD values across the 20-confomers-ensemble (0.38 Å for backbone atoms and 0.85 Å for heavy atoms). Within the folded region, 83% of the side chains of residues different from Ala, Gly and Pro are ordered (RMSD≤±30° for the dihedral angle χ1) and ten of them are also well-ordered at level of angle χ2.

**Table 1:** Summary of NMR restraints and structural statistics for the ensemble of 20 lowest energy structures of Pub1p R3 (residues 315–414).

**Distance restraints**	
Intraresidue	277
Sequential	440
Medium range (2≤|i−j|≤4)	421
Long range (|i−j|≥5)	830
All	1968
*Averaged NOE violations per structure (Å)*	*0.0045±0.0005*
**Dihedral angle restraints**	
φ	61
ψ	68
All	129
*Averaged dihedral angle violations per structure (deg)*	*0.32±0.08*
**Averaged pair-wise RMSD (Å)**	
Backbone atoms (324–412)	0.38±0.08
All heavy atoms (324–412)	0.85±0.10
**Ramachandran plot**	
Residues in most favoured regions	76.4%
Residues in additional allowed regions	21.7%
Residues in generously allowed regions	1.2%
Residues in disallowed regions	0.6%

Pub1p C-terminal domain adopts a typical α/β RRM-type fold with a β_1_α_1_β_2_β_3_α_2_ topology [5], [6], [7], [45], but with some key features in comparison with other RRM domains. First, the β-sheet is distorted in Pub1p R3, and the last strand β_4_ is missing. Instead there is a short β-hairpin (400–405) right after the helix α_2_ (386–396), previously reported in other RRM structures [7] and, more importantly, a non-canonical N-terminal α-helix (labelled here as α_0_: 326–333). This novel helix is stabilised by extensive contacts with helix α_2_ and the C-terminal residues (Figure 2C), chiefly Trp 410, which is at the centre of a hydrophobic cluster (Val 329, Ile 332, Ile 333, Ala 336, Pro 337, Val 340, His 386, Ala 390 and Ile 393) that defines the orientation of the new helix. The side chain imino proton of this residue forms a H-bond with the carbonyl group of Ile 332 that drives Trp 410 out of its normal position as part of the β-sheet, causing a local distortion of it and hindering the formation of the fourth strand. Consistent with its structural role, Trp 410 is strictly conserved across different fungal Pub1p proteins (Figure 2D), Ngr1p, Nam8p (two Pub1p homologues in yeast), TIA-1 and TIAR proteins. The sequence alignment along these proteins allowed us to identify a conserved motif (Trp-Gly-[Arg/Lys]) which is the hallmark of the family.

We pursued a thorough study of the role of Trp 410 by analysing the properties of a set of 11 mutants including replacements by aromatic (Tyr, Phe and His), hydrophobic (Leu, Ile and Pro), polar (Asn, Ser, Thr) and small (Ala and Gly) side chains. All mutants exhibited poor solubility when over-expressed at 37°C, a situation that is partially alleviated when expressed at 20°C (Figure S1A), however the correlation with the amino acid type is unclear. The poor solubility hindered the isolation and purification of these proteins and some of the soluble ones at low temperature turned to be highly unstable during the purification (i.e. Trp410Ile). We were only able to obtain enough quantities to perform further biophysical studies for the Trp410Phe mutant. The comparison of the melting curves of the wild-type and mutant proteins followed by circular dichroism shows that the later is about 10°C less stable (Figure S1B). The large decrease in ΔH_m_ together with the more shallow profile of the curve is indicative of a less co-operative unfolding mechanism for the mutant, perhaps attributable to the absence of the N-terminal helix. The overall conclusion of this study is that the Trp 410 position is critical for protein solubility and stability. The buried hydrogen bond Trp 410 Nε_1_-Ile 332 CO seems to be the key interaction that enforces a tryptophan side chain at this position.

To summarize: the C-terminal (Trp-Gly-[Arg/Lys]) motif, the distorted three-stranded β-sheet and the new N-terminal helix represent novel features that are likely conserved in the RRM3 domains of Pub1p/TIA-1 proteins and represent the hallmarks of a new class of RRMs that we propose to name TRRM (TIA-1 C-terminal domain like RRM).

### Pub1p R12 recognises poly(U) with high affinity and specificity

**Figure 3:**
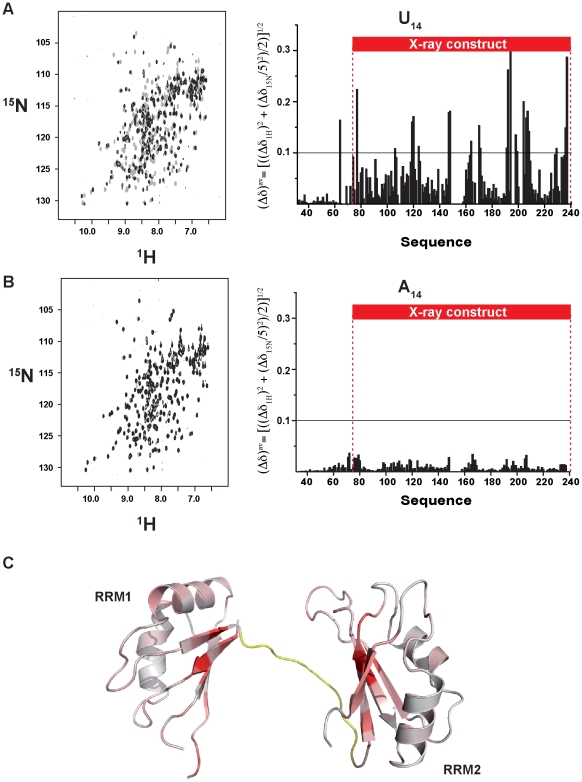
RNA recognition by Pub1p R12. (**A**) Superposition of the ^1^H-^15^N HSQC spectra of the free Pub1p R12 protein (100 µM; in black) and the complex with U_14_ (100 µM each, gray) (left). Histogram showing the weighted averaged ^1^H and ^15^N amide chemical shift changes, Δδ^av^ (right). (**B**) Superposition of the ^1^H-^15^N HSQC spectrum of Pub1p R12 (100 µM) in the absence (black) and in the presence (gray) of 100 µM A_14_ (left). Histogram displaying the averaged ^1^H and ^15^N amide chemical shift changes for this complex (right). Assignments of the residues 149–157 are missing for the free protein and for the complexes. (**C**) Red color-coded mapping of chemical shift changes observed upon U_14_ titration on the X-ray structure of Pub1p RRM1-RRM2 [43] (PDB: 3MD3). Unassigned residues in the linker between RRM1 and RRM2 are colored in yellow.

Pub1p binds poly(U) sequences *in vitro* [3] and we decided to investigate which regions of the protein are responsible for this activity and specificity. First we explored the RNA binding of the Pup1p R12 construct by NMR titration experiments (Figure 3). We tested three different probes: U_14_, A_14_ and (UA)_7_, which have very similar composition to that of the RNA motifs found in the UTRs of Pub1p-bound mRNAs [15]. The U_14_ probe binds with high affinity in the slow-exchange regime, a behaviour typical of protein-RNA complexes with dissociation constants up to 250 nM [46]. Therefore, our data demonstrate that the first two RRMs are sufficient to account for the polyuridine binding activity of Pub1p [3]. In contrast, A_14_ probe titrates in the fast chemical shift exchange regime, typical of protein-RNA complexes with affinity higher than 15 µM [46]. However the chemical shift perturbations are negligible (Figure 3) at equimolecular ratio (100 µM protein∶RNA), indicating that the bound form is scarcely populated at the working concentration (i.e. K_D_>100 µM). Finally, the (UA)_7_ probe titrates in the intermediate chemical shift time scale which is characterized by signal disappearance (by exchange broadening) upon titration and even at stoichoimetric conditions. This behaviour is typical of protein-RNA complexes with K_D_ ranging between 400 nM and 2 µM [46].

Mapping of chemical shift perturbations (CSP) onto the X-ray structure of Pub1p RMM1-RMM2 construct (residues 75–240) [43] reveals that both RRM participate in RNA recognition (Figure 3C). In this case, unlike other well-known examples [47], [48], [49], [50], it is not clear whether the linker region participates in the interaction, as the assignment of these residues remains elusive in the complex. The largest changes are concentrated on the canonical recognition interfaces (β-sheets), hence we might expect a recognition mode similar to other RRM-containing proteins that bind polyuridine tracks [47], [49], [51], [52]. Strikingly, the CSP effect propagates beyond the boundaries defined for the X-ray construct as we could detect RNA-dependent changes up to the residue Ala 63 (Figure 3A). This evidences the importance of intrinsically unstructured regions like this (residues 63–74) in ligand binding. Remarkably, Pub1p fungal orthologues show a totally conserved tetrapeptide motif (GGRE in positions 69–71) in this region. Although the way it participates in protein-RNA recognition remains as an open question.

### Pub1p R3 recognises U-rich and UA-rich RNAs preferentially

**Figure 4:**
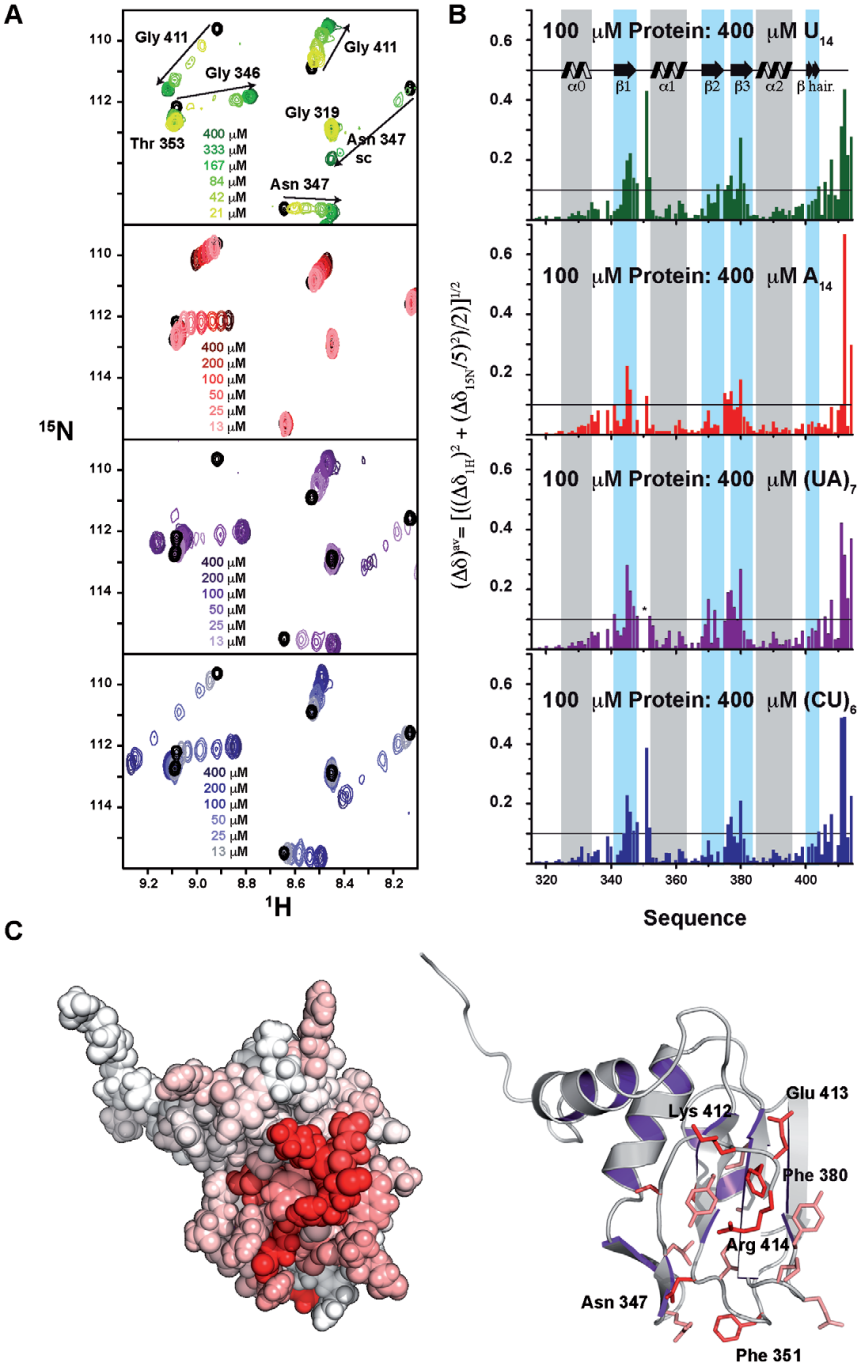
RNA recognition by Pub1p R3. (**A**) Superposition of a selected region of the ^1^H-^15^N HSQC spectra of the Pub1p R3 domain in the absence of ligand (black) and in the presence of increasing amounts of U_14_ (green), A_14_ (red), (UA)_7_ (purple) and (CU)_6_ (blue) RNA ligands. Resonance assignments are indicated on the top spectrum. All the spectra were acquired at 100 µM protein concentration in 0.5 mM DTT, 25 mM NaCl, 25 mM potassium phosphate buffer (pH 7.0) and 25°C. (**B**) Averaged ^1^H and ^15^N amide chemical shift changes (Δδ^av^) observed in the ^1^H-^15^N HSQC spectrum of Pub1p R3 (100 µM) in the presence of a 4-fold excess of the corresponding RNA target (color code matching that of panel A). An asterisk indicates that Phe 351 resonance broadens out beyond detection upon (UA)_7_ binding. Secondary structure elements are depicted on the top graph. (**C**) CPK (left) and cartoon (right) representations of the Pub1p R3 NMR structure. Residues have been shaded in red range according their Δδ^av^ values (from white = 0.0 to bright red = Δδ^av^_max_) for the titration point with 400 µM U_14_ (upper panel in B). Only side chains of amino acids with Δδ^av^≥0.1 ppm are shown onto the cartoon image for clarity. The figures were generated with the program PyMOL v0.98 (DeLano Scientific LLC, Palo Alto, CA, USA).

We also investigated RNA binding by Pub1p R3 through ^1^H-^15^N HSQC-based NMR titration experiments with several RNA probes (Table 2). Pub1p R3 forms complexes in the chemical shift fast exchange regime for uracyl-free RNA probes (i.e. A_14_, (AG)_4_, (AC)_4_ and C_8_) and in fast exchange with line broadening, or “insufficient fast exchange” [53], for uracyl-containing RNAs [U_10_, U_12_, U_14_, (UA)_6_, (UA)_7_ and (CU)_6_] (Figure 4). The first group of complexes display binding affinities around 70 µM or weaker (Table 2). K_D_ values were calculated by a global fitting of the NMR signals with Δδ^av^>0.1 ppm (Equation 1) with a non-linear least squares protocol. The same fitting procedure was followed for the second group of RNA probes but excluding the crosspeaks experiencing “insufficient fast exchange” for which equation 2 (see Materials and Methods) can not be applied [53]. The U-rich (U_14_, U_12_, U_10_) and UA-rich series [(UA)_7_, (UA)_6_] display K_D_ values around 1-order of magnitude tighter than (CU)_6_ and at least two-orders tighter than the A-rich sequences of the first group (Table 2). Furthermore we found that U_14_ and (UA)_7_ form 2∶1 protein-RNA complexes whereas U_12_ and (UA)_6_ form 1∶1 complexes. Thus, we can conclude that Pub1p R3 binds an RNA stretch no longer than 6 bases, close to the longest ssRNA bound by an isolated RRM domain [54].

**Table 2:** Intervals of K_D_ values^a^ for binding of RNA oligonucleotides to Pub1p R3 calculated at the 95% confidence level.

RNA	K_D_ (µM)	Stoichiometry (Protein∶RNA)
U_14_	1.2–3.9	2∶1
U_12_	n.d	1∶1
U_10_	n.d	1∶1
(UA)_7_	<1.0	2∶1
(UA)_6_	n.d.	1∶1
A_14_	70–88	2∶1
(CU)_6_	16–22	1∶1
(GU)_4_ b	n.d.	n.d.
(AG)_4_	78–100	1∶1
(AC)_4_ c	very weak	1∶1
C_8_ c	very weak	n.d.

a All the resonances showing Δδ^av^>0.1 ppm upon RNA binding were used in the data fitting to derive K_D_ values.

b The K_D_ values could not be determined due to precipitation of the complex during the titration experiments.

c The K_D_ values could not be determined due to incomplete saturation of the protein signals.

n.d. not determined.

The three-dimensional distribution of the RNA-induced CSP is similar for all the RNA probes (Figure 4B), with the exception of Asn 347 side chain (Figure 4A). The Nδ2-Hδ21 and Nδ2-Hδ22 peaks suffer variations between 0.2 and 0.4 ppm for U_14_, (UA)_7_ and (CU)_6_ complexes, but hardly any change for the A_14_ one. Residue Asn 347 is in the β_1_-α_1_ loop next to Phe 351, that also changes in the uracyl-containing RNA probes. Hence we propose that this loop contains an uracyl recognition hot spot in the protein, with Asn 347 side chain probably making specific contacts with the base moiety. A hydrogen bond between Asn 347 Hδ2× and the O4 of the uracyl would explain base specificity at this position, with Phe 351 making stacking interactions to the base. Interestingly, neither Asn 347 nor Phe 351 are conserved in TIA-1 proteins (Figure 2D), thus a different RNA selectivity might be expected for these proteins. The C-terminal tetrapeptide Gly 411-Lys 412-Glu 413-Arg 414 experiences very large CSP in the complexes and are surely involved in RNA binding (Figure 4B and 4C). The remaining changes are localized in the β-sheet, the usual RNA docking platform in most RRMs [7]. Interestingly, neither Trp 410 nor α_0_ helix residues suffer significant perturbations, thus we can conclude that this novel element is not participating in RNA recognition.

Pub1p R3 binds (UA)_7_ probes with low micromolar affinity, which is the typical affinity range displayed by single RRM domains recognising single strand RNA fragments with some degree of specificity [51], [55], but still weaker than those displaying a high degree of specificity [54]. The recently published structure of CUG-BP1 RRM3 [55] is probably a close model of Pub1p R3 binding mode, as this protein recognises a related sequence (UG)_3_ with similar affinity (K_D_ = 1.3 µM) than Pub1p R3.

In conclusion, we found that Pub1p contains two separated RNA recognition platforms (RRM1-RRM2 and RRM3) which bind U-rich and UA-rich sequences with higher affinity than A-rich ones. The recognition of 3′-UTR by Pub1p might be commanded by the N-terminal region (which binds RNA U-stretches with greater affinity) with the C-terminal one playing an auxiliary role in the selection of the transcripts.

### Pub1p RRM3 interacts with Box1 region of Tif4631p (eIF4G)

**Figure 5:**
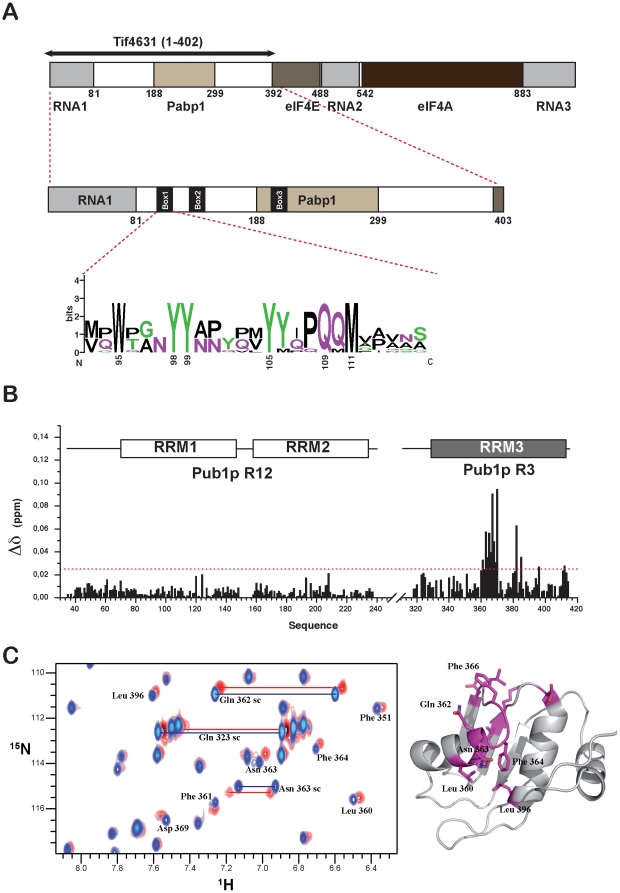
Monitoring Tif4631p recognition on the Pub1p NMR spectra. (**A**) Domain organization of Tif4631p (up) displaying recently described Box1, 2 and 3 in the N-terminus of the protein [66]. Schematic representation of the largest Tif4631p construct used in this study (middle) and pattern of sequence conservation of Box1 region in different *Saccharomyces* species [66]. (**B**) CSP of Pub1p R12 and Pub1p R3 backbone amide resonances (combined ^1^H and ^15^N values) caused by Tif4631p (1–402) titration (in an ∼2∶1 excess). Data sets were recorded independently for both Pub1p constructs at similar concentration and protein-protein ratios. (**C**). Comparison of the free and Tif4631p-bound Pub1p R3 ^1^H-^15^N HSQC spectra in the region of the Gln and Asn side chains (left). Chemical shift mapping of the Tif4631p binding site (right), with residues changing more than 0.025 ppm depicted in magenta.

**Figure 6:**
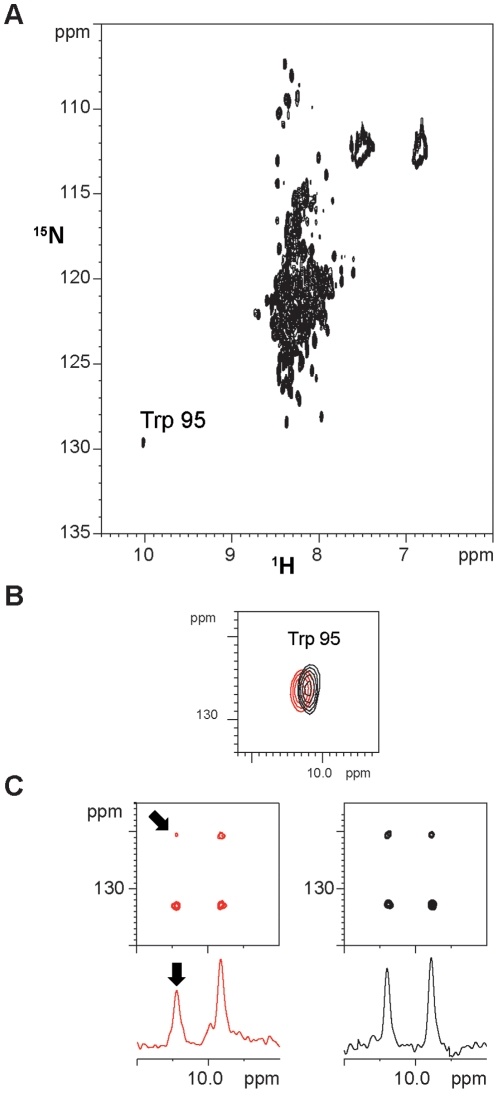
Monitoring Pub1p recognition on the Tif4631p NMR spectra. (**A**) ^1^H-^15^N HSQC spectrum of the construct Tif4631p (1–402) showing the characteristic low dispersion of an intrinsically unstructured protein. Signal corresponding to the side chain of Trp 95 has been labelled. (**B**) Chemical shift perturbation on the Trp 95 Hε1-Nε1 crosspeak: free Tif4631p protein in black and a complex with Pub1p R3 in red (see Materials and Methods for details). (**C**) Analysis of line shape variations caused by Pub1p binding in the multiplet components of the Trp 95 Hε1-Nε1 signal. Coupled ^1^H-^15^N HSQC (up) and 1D (down) spectra have been included for comparison. The low field components of the multiplet experience further line broadening in the spectra of the bound form (marked with arrows) in comparison with that in the free form (black). This effect is linked to a reduction in mobility due to the complex formation (see text for further details). Tif4631p concentration and buffer conditions are identical in both free and bound spectra, which have acquired with same parameters and represented at equal contour levels.

The TRRM domain has been exclusively found in Pub1p, TIA-1 and TIAR proteins, suggesting that it may play a unique role in the biological function of this family of proteins. Proteomic studies have identified Pub1p-binding proteins, which includes eIF4G yeast homologues Tif4631p, and Tif4632p [56], [57]. The Tif4631p/Pub1p interaction has not been studied in detail yet, however it has been proposed to be involved in translation control of the MFA1 transcript in response to carbon source changes [19]. Additionally, the Pub1p and Tif4631p are essential components of the EGP-bodies generated upon glucose starvation [29], robust heat shock stress granules [31] and their homologues (TIA-1/eIF4G) are constituents of mammalian stress granules [21], [22], [26], [58], [59]. Tif4631p is a scaffold protein with a C-terminal part that contains eIF4A- and eIF4E-interacting domains (Figure 5A). Structures of these complexes have been reported [60], [61]. Much less is known for the N-terminal part that is predicted to be intrinsically unstructured and includes the Pabp1 binding region [62]. We overexpressed a Tif4631p construct corresponding to this region (residues 1–402), obtaining a soluble protein that, as previously shown [63], migrates at apparently higher molecular weight in the SDS-PAGE gels. Nevertheless, the protein identity was confirmed by mass spectroscopy and the recombinant protein shows the typical ^1^H-^15^N HSQC NMR spectrum of an intrinsically unstructured protein, sharp and very low dispersed signals (Figure 6). However, the existence of regions with some local secondary structure propensity cannot be entirely ruled out. First, we tested if Tif4631p (1–402) interacts with any of our Pub1p constructs by ^1^H-^15^N HQSC titration experiments, mixing unlabelled Tif4631p protein with ^15^N-labelled Pub1p R3 or Pub1p R12 in approximately 2∶1 molar ratio. The comparison of the CSP for the two Pub1p constructs (Figure 5B) showed that Tif4631p (1–402) interacts specifically with Pub1p TRRM domain. The magnitude of the CSP is small but non-randomly distributed, suggesting that the interaction is weak but specific. Similar weak but specific effects are observed in the NMR titrations in the human eIF4A/4G/4H helicase complex [64]. The Tif4631p binding site is formed by residues of strand β_3_, helix α_1_ and the linker between them (Figure 5C) and includes conserved Phe 364 and Phe 366 (in fungi). The side chain amide groups of Gln 362 and Asn 363 show significant CSP upon binding (Figure 5C), maybe because make specific interactions across the interface (i.e. hydrogen bonds). Among these four residues only Phe 364 is conserved in TIA-1, thus if an equivalent interaction with eIF4G exists it is likely to be topologically different.

Next, we analysed the perturbations on the Tif4631p NMR spectra by mixing unlabelled Pub1p R3 in a 2∶1 molar excess with ^15^N-labelled Tif4631p (1–402) (Figure 6). This approach provides a complementary view of the interaction by showing the effect on Tif4631p. The high overlapping of Tif4631p ^1^H-^15^N HSQC spectrum masks most of the expected changes, although the lower-left crosspeak of the spectra shows changes upon binding (Figure 6B). Because of its unique position, this signal could luckily be assigned to Trp 95 Nε1-Hε1, the only Trp residue of Tif4631p (1–402). This evidences that Pub1p R3 either contacts this residue or at least is in its vicinity. Even more, the CPS changes are accompanied by differential changes in the signal line widths of the individual components of the Nε1-Hε1 multiplet. In the free state all the four signals showed similar intensity and line widths, a situation typical of a highly mobile group (i.e. intrinsically disordered protein) (Figure 6A). However, upon binding, all the components suffer line broadening, which is higher for the left components of the multiplet. This differential effect is attributable to the interference of NMR relaxation mechanisms (dipole-dipole and chemical shift anisotropy), popularly known as the TROSY effect [65]. This change on the relaxation properties of the Trp signal undoubtedly shows that this side chain experiences a reduction in mobility attribuitable to the association with Pub1p R3. NMR data, CSP and the changes in line width, demonstrate that Pub1p R3 binding site overlaps with Tif4631p Box1 (Figure 5A), a highly conserved region in *Saccharomyces* eIF4G homologs [66] close to the Pab1p binding site.

**Figure 7:**
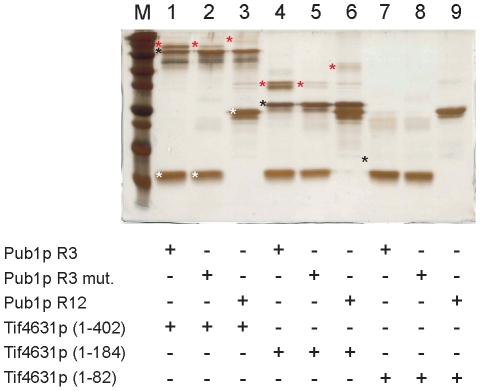
Biochemical *in vitro* cross-linking experiments probing the interaction between Pub1p and Tif4631p. The potential interaction among different combinations of Pub1p and Tif4631p constructs has been assayed by cross-linking with glutaraldehyde using a vapour diffusion method (see Materials and Methods for details). The silver-stained 15% PAGE gel shows the protein composition of different cross-linking reactions: Pub1p and Tif4631p constructs have been marked with white and black asterisks and detected adducts with red ones. Construct Tif4631p (1–82) was inefficiently stained with silver (lines 7,8 and 9), although its approximate position of (as seen in coomassie stained gels) has been included for reference. Minor bands observed bellow the Tif4631p (1–402), Tif4631 (1–184) and Pub1p R12 ones, correspond to intramolecular cross-linking reactions and/or degradation products. Equivalent bands are found in the control experiments (Figure S3).

To complement the NMR analysis, we decided to perform independent biochemical experiments. First, we chose affinity capture experiments by GST tagging either Tif4631p or Pub1p constructs, but we faced technical problems due to resin-induced protein precipitation for some Pub1p constructs, that interfere with the outcome of the experiment. As an alternative, we performed *in vitro* protein-protein cross-linking experiments using glutaraldehyde. This time, the large number of potentially reactive groups in Tif4631p (1–402) (48 Lys side chains) resulted in a large amount of unspecific reactions (self cross-linking mainly). We optimized the reaction by keeping protein concentrations below 10 µM and adjusting the Tif4631p/Pub1p ratio to the ratio of Lys on each protein (see Materials and Methods for further details). We also followed an adaptation of a vapour diffusion method that ensures a slow addition of the cross-linking agent [67] and minimizes unspecific reactions. The formation of the corresponding adducts was monitored by PAGE-SDS. Three different Tif4631p constructs were used: the original Tif4631p (1–402) and two shorter forms Tif4631p (1–184) and Tif4631p (1–82). On the other side, we used the Pub1p R12 and Pub1p R3 constructs and a double mutant of Pub1p R3 Phe364Ala/Phe366Ala that targets key residues of Tif4631p binding site identified by NMR. The results are summarized in Figure 7 and confirm the conclusions obtained from the NMR analysis. Tif4631p (1–402) and (1–184), that contain Box1, form adducts with Pub1p R3, but not with Pub1p R12 or with Pub1p R3 double mutant. The lost of binding in the mutant is highly revealing as demonstrates the direct involvement of Phe 364 and Phe 366 in the interaction. An alternative explanation arguing that mutations cause Pub1p R3 unfolding could be plausible, but the high resemblance between Pub1p R3 mutant and wild-type NMR spectra show that their structure must be similar (Figure S2). Unfortunately, Tif4631p (1–82) can not be stained with silver (Figure S3B, lines 3 and 6) and is only visible by negative staining on overexposed silver stain gels (data not shown). Nevertheless, none of the Pub1p variants form adducts with this construct, showing that the so-called RNA1 region in Tif4631p [66] (Figure 5A) does not interact with Pub1p.

In conclusion, our study about the molecular recognition properties of Pub1p shows a very versatile protein capable to recognise Tif4136p and RNA using structurally compatible interfaces (Figure 4C and 5C). However, there are novel structural features discovered, such as the conserved N-terminal helix in Pub1p R3, whose function is still poorly understood, opening new avenues for future research.

## Discussion

### The novel C-terminal RRM domain is conserved in Pub1p/TIA-1 protein family

ELAV-like HuR and TIA-1/TIAR proteins have been proposed to be the metazoan homologues of Pub1p on the basis of its similar functional role in mRNA metabolism. However, the structure of Pub1p R3 shows novel sequence features (the Trp-Gly-[Arg/Lys] motif), which are present in TIA-1 proteins but not in HuR ones, supporting that TIA-1/TIAR are the true evolutionary relatives of Pub1p. An unpublished structure of the third domain of TIAR protein has been deposited in the PDB data bank (ID 1X4G) and it also contains the novel N-terminal helix constituting, together with our structure, the only two cases of RRMs having this non-canonical secondary structure element. We propose to name this new class of domains as TRRM (TIA-1 C-terminal domain like RRM). The conservation of this structural feature might be correlated with a biological function common in TIA-1 and Pub1p proteins. In metazoans, TIA-1 is involved in stress granules formation [22], [34], [58] and in alternative splicing [37], [40], [41], [42]. In yeast, Pub1p and Ngr1p (TIAR homolog) are components of different cytoplasmic RNA granules (recently reviewed in [26]) while Nam8p is involved in RNA splicing [38], [68]. Although the level of conservation of the Trp-Gly-[Arg/Lys] motif is paramount among the Pub1p/TIA-1 family, Ngr1p and Nam8p sequences lack the characteristic amphipatic profile in the region corresponding to the hypothetical N-terminal helix (Figure 2D), suggesting that this element is not present in these proteins. We have attempted to overexpress the TRRM domain of Ngr1p but it proved to be highly insoluble (data not shown), maybe because the absence of the N-terminal helix exposes the hydrophobic cluster beneath it. Similarly the destabilization of the same region in the Pub1p Trp410 mutants (Figure S1) could also decrease the solubility, perhaps due to a similar effect.

Pub1p is not the only RNA binding protein found in cytoplasmic RNA granules that contain non-canonical RRM fold. The second RRM of TDP-43, a component of some types of mammalian stress granules [69], [70], [71], has an additional β-strand in the β-sheet), which is involved in protein dimerization [72]. The TDP-43 dimer interface includes helix α1 and self-recognition is guided by hydrogen bonds. It could be possible that Pub1p novel N-terminal helix functions similarly, however we have not found evidences by NMR, gel filtration and analytical ultracentrifugation of such behaviour. Moreover, the cross-linking experiments, which are very efficient in capturing transient interactions, did not detect the presence of a Pub1p TRRM dimer (compare lines 1 and 4 in Figure S3A).

### Pub1p R3 binds Tif4631p using a similar interface to other protein-binding RRMs

**Figure 8:**
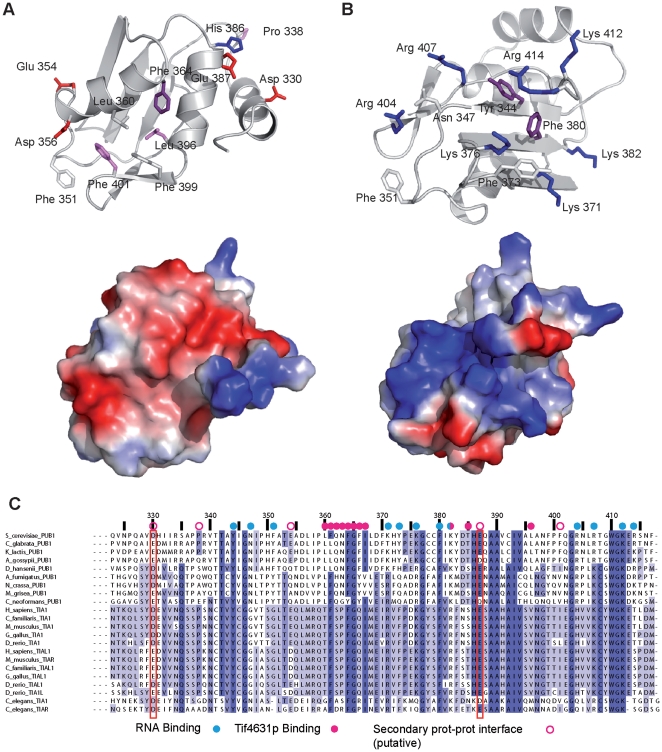
Comparison between RNA and protein recognition interfaces of Pub1p R3. (**A**) Conserved hydrophobic (Phe 364, Leu 396 and Phe 401 in purple) and acidic residues dominate a large negatively charged protein-protein interface. The extended interface is constructed by the three helices of the structure. Additional hydrophobic residues (Phe 399, Phe 351 and Leu 360) define a hydrophobic patch in the helix α1–α2 interface. Conserved acid side chains (Glu 387 and Asp 330) characterize the helix α0–α2 interface. See text for further details. (**B**) In contrast, the RNA binding interface is dominated by basic residues and aromatic side chains. The number of conserved Arg/Lys side chains is similar to the number of oligonucleotides (and hence phosphate groups) likely bound by Pub1p R3. Base moieties might form stacking interactions with the aromatic groups present in this surface. (**C**) Sequence alignment of the TRRM domains of Pub1p/TIA-1 proteins (sequence numbers correspond to *S. cerevisiae* Pub1p). Aminoacids positions are highlighted in different shades of blue according to their percentage of identity. Conserved acid residues are marked with a red rectangle. Residues involved in RNA and Tif4631p binding interfaces are coded by the corresponding symbol above the sequence.

A detailed structural analysis, in combination with the alignment of TIA-1/Pub1p TRRM sequences, identifies conserved residues in the opposite face to the RNA interface (Figure 8). The backside of Pub1p TRRM is shaped by the contacts between the three helices of the structure, which define a negatively charged interface with two grooves and three ridges (Figure 8). Two conserved residues (Asp 330 and Glu 387) characterize the shallow groove (not present in previous RRM structures) defined by helix α_0_ and helix α_2_; with the hydrophobic Pro 338 placed at the bottom of the imaginary valley. His 386 side chain, also in the region, is allowed to change to Arg or Lys, conserving a positive character that seems to be key for the π-cation interaction with Trp 410 and hence for protein stability (Figure 2C). The second groove, defined by helix α_1_ and helix α_2_, is wider and deeper and contains conserved hydrophobic side chains (Figure 8) and one or two negatively charged side chains on helix α_1_. An structurally equivalent region is involved in recognition of Trp-containing peptides in U2AF homology motifs (UHM), a well-known family of RRMs capable to interact with proteins and RNAs simultaneously [73] (Figure S4). In a more recent example, the same groove is used for protein FIR to recognise an α-helical segment of FUS through a novel protein-protein binding mode [74] (Figure S4). Like in the later case, Tif4631p interacts with Pub1p TRRM by using the α_1_/α_2_ groove, but also the linker connecting α_1_ and β_3_ and part of β_3_, suggesting a possible novel mechanism of RRM-mediated protein recognition. Our study identifies Box1 as the Pub1p interacting region in Tif4631p. This peptide motif contains a totally conserved tryptophan in *Saccharomyces* eIF4G homologs ([66] and Figure 5A), therefore the interaction might partially resemble that of the UHM. Interestingly, Pabp1 does interact with Tif4631p through its RRM2 domain [9], [75], using an interface similar to that of Pub1p TRRM. In this case, Pab1p targets the conserved peptide Box3 in Tif4631p, which neighbours the Pub1p binding site (Figure 5A). The structure of the Pab1p/Tif4631p complex has not been solved, however the recognition of mammalian eIF4G1 by the rotavirus NSP3 protein, a functional homolog of PABP1, provides solid evidences on the binding mode [75]. Protein-protein recognition would involve two conserved residues in eIF4G1 (Ile 140 and Ile 142) that presumably pack against Phe 119 and Phe 122 of PABP1 RRM2. Equivalent residues can be found in Pab1p RRM2 and Tif4631p Box3 suggesting that the binding mode is conserved across species. We have experimentally proved that Pub1p Phe 364 and Phe 366 (structurally equivalent to PABP1 Phe 119 and 122) are involved in recognition (Figure 5C). Tif4631p Box1 does not contain conserved Ile residues, but rather has a series of aromatic side chains (Figure 5A) that would presumably participate in Pub1p binding. All these observations suggest that Pub1p and Pab1p share a similar Tif4631p recognition mechanism. Giving the high level of conservation of Box1 in Tif4632p, it is reasonable to think that Pub1p recognises it in a similar fashion.

### Determinants of Pub1p recruitment to the mRNPs

According to chemical shift perturbation data, Pub1p TRRM might be able to recognise Tif4631p and RNA specifically and simultaneously. The Pub1p-binding box in Tif4631p is close to the Pab1p binding site, suggesting the possibility that both proteins could work cooperatively to recognise more than one sequence motif in the UTRs. Alternatively, it could be possible that Pub1p recruitment to the mRNPs was commanded by the interaction with Tif4631p, in an RNA-independent manner. We find interesting to discuss about the biological implications of these new structural findings and to see the degree of agreement with current models about different aspects of mRNA metabolism. Pub1p is loaded to the nuclear mRNPs and then shuttled to the cytoplasm, where is part of the fraction of translationally inactive mRNPs [3]. The latter suggests that the protein is probably removed from the mRNPs at some stage of the translational initiation [13], or perhaps later during the pioneer round of translation [76], although this issue has not been yet experimentally addressed. Different types of stressors cause the interruption of the normal translation initiation flow path, causing the accumulation of pre-initiation complexes (PIC) that eventually aggregate into different types of cytoplasmic granules. It is known in yeast and mammals that Pub1p/TIA-1 proteins are components of some of these granules ([26] and references herein). In mammals, the most likely mechanism of TIA-1 mediated SG nucleation involves its C-terminal prion-like region [34], [36]. Pub1p also contains an equivalent C-terminal region and additionally a Met/Asn-rich region between RRM2 and RRM3 that could play a similar role. The accumulation of stalled PICs at a stage that they still contain TIA-1/Pub1p, would trigger their aggregation into RNA granules (e.g. EGP-bodies or SG) above a certain concentration threshold.

Pub1p promotes stabilization of mRNAs against ARE-mediated deadenylation decay and NMD pathways, which occur in the P-bodies. Thus, it is likely that Pub1p binding prevents (or delays) the localization of a subset of mRNPS into P-bodies. Hence it seems important to unravel which are the determinants of Pub1p recruitment to these transcripts. A genome-wide study identified 368 cellular transcripts bound to Pub1p and, among them, 53 whose stability is decreased in the absence of Pub1p [15]. Most of these mRNAs encode proteins that act in ribosomal biogenesis and cellular metabolism. Furthermore, after a re-examination of these data we have found that the Pub1p-bound dataset is enriched in transcripts derived from intron-containing genes (48 out of 368). Surprisingly the percentage increases up to 40% in the 53-subset (transcripts which are simultaneously bound and stabilized by Pub1p), suggesting that splicing modulates Pub1p-mediated stabilization, probably by promoting its recruitment to the nascent transcript. TIA-1 is a component of metazoan spliceosome [37], [39], [40], [41], [42] but this biological function is performed (at least partially) by Nam8p in yeast [38], rather than by Pub1p. We propose three possible mechanisms of Pub1p recruitment to the nascent mRNP in a splicing-dependent manner: binding to known spliocesome components (i.e. Lms2p, Lms4p, Sto1p, Tif4631p) [77], replacement of Nam8p and specific recognition of RNA trans-acting elements in introns or exon junctions (typically U-rich tracks).

Apart from the splicing-dependent mechanism there should be an alternative Pub1p recruitment mechanism to account for transcripts derived from intronless genes. We agree that such procedure might depend on the cooperative recognition of RNA regulatory elements in the UTRs [15]. A-rich, U-rich and UA-rich motifs are overrepresented in the 3′- and 5′-UTRs of Pub1p-bound mRNAs [15]. Since Pub1p has low affinity for A-rich oligonucleotides, these authors proposed that additional RNA-binding proteins could act in coordination with Pub1p to achieve A-rich recognition. If Pub1p interacts with Tif4631p *in vivo* through a similar mode to that described here, transcripts containing A-rich and U-rich motifs could be recognised in a cooperative manner by Pub1p and Pab1p thanks to the simultaneous interaction of both proteins with neighbouring sites in Tif4631p. The model does not exclude the participation of other RBPs shuttle proteins like Hrp1p, Npl3p, Hrb1p or Gbp2p, some of which can be tethered to eIF4G, in a similar way than Pub1p and Pab1p does, to Tif4631p.

## Materials and Methods

### DNA cloning and site-directed mutagenesis

DNA fragments corresponding to wild-type constructs were amplified from *Saccharomyces cerevisiae* genomic DNA (Novagen) by PCR using high fidelity DNA polymerase KOD (Novagen) and specific DNA primers (Sigma, IDT). The products were purified, digested with the corresponding restriction enzymes and ligated into a home-modified pET28 (Novagen) vector that contains: an N-terminal thioredoxin A fusion tag, for enhanced expression and solubility, an internal 6×His tag for affinity purification and a TEV protease site, for fusion tag removal. Three constructs of *S. cerevisiae* Pub1p protein were obtained: Pub1p R13 that spans over three RRM domains (residues 32–414), Pub1p R12 containing domains RRM1 and RRM2 (residues 32–242) and Pub1p R3 that comprises domain RRM3 (315–414). The three *Saccharomyces cerevisiae* Tif4631p constructs (residues 1–402, 1–184 and 1–82) were cloned following equivalent methods.

The Trp410 library of mutants was generated in the Pub1p R3 framework by using the QuickChange site-directed mutagenesis® kit (Stratagene) and a DNA primer with a random base composition of codon 410 (5′-G GTG CTC GAG TTA TCT TTC CTT ACC RNN ACC GGT TCT CAA GTT TC-3′; R = A or G and N = A,G,C or T). Pub1p R3 Phe364Ala/Phe366Ala double mutant was generated using an equivalent protocol.

### Protein expression and purification

Plasmids corresponding to wild-type and mutant proteins were transformed in *E. coli* BL21(DE3) (Novagen) chemically competent cells and expressed in kanamycin (Sigma-Aldrich) containing (30 µg/l) LB medium. For isotopic labelling, a K-MOPS derived minimal medium [78] was supplemented with ^15^NH_4_Cl (1 g/l) and/or ^13^C-glucose (4 g/l). Bacterial cultures were typically grown at 37°C until OD_600_ = 0.6, transferred to a shaker at 20°C and induced overnight with 0.5 mM IPTG (Sigma-Aldrich) upon temperature stabilization. Cells were harvested by centrifugation (30 minutes at 3000 g), resuspended in buffer (20 mM potassium phosphate pH 8.0, 300 mM NaCl, 0.1% β-mercaptoethanol) containing protease inhibitors (Roche) and lysed by French-press (Thermo Scientific) or sonication. Lysates were pelleted at high speed (30 minutes at 15000 g) to separate cell debris and other insoluble components. Wild-type protein constructs (Pub1p R12, Pub1p R3 and Tif4631p) and Pub1p mutants were first purified by metal affinity chromatography (HisTrap™ HP 5 ml (GE Healthcare)). Fractions coming from this column that contain the fusion protein were pooled together, dialysed (overnight) at 4°C against 20 mM potassium phosphate pH 8.0 buffer (plus 0.1% β-mercaptoethanol) and simultaneously digested with homemade TEV protease (100–200 µg/ml). After complete digestion, the samples were re-applied to the nickel column and the target proteins were collected in the flow through. In the final step, proteins were purified and concentrated by ion-exchange chromatography: Pub1p R12 and Tif4631p with an anion exchange column (MonoQ 5 ml, GE Healthcare) and Pub1p R3 with a cation exchange one (SP 5 ml, GE Healthcare). In either case, proteins were eluted with a linear salt gradient (from 0 to 1 M NaCl) in a buffer containing 20 mM Tris-HCl pH 8.0 and 1 mM DTT. Pub1p mutant proteins were purified with similar methodologies than wild-type proteins. Sample purity and homogeneity was checked by PAGE-SDS and mass spectrometry.

### NMR spectral assignment and structure calculation

Protein samples for NMR spectroscopy were approximately 500–800 µM for Pub1p R12 and 300 µM for Pub1p R3 in 90% H_2_O, 10% D_2_O, 0.1% sodium azide containing 25 mM potassium phosphate buffer (pH 6.5–7.0), 25 mM NaCl, and 0.5 mM DTT. All experiments were recorded in Bruker AV600 and AV800 NMR spectrometers equipped with cryoprobes. Sodium 2,2-dimethyl-2-silapentane-5-sulphonate (DSS) was used as an internal chemical shift reference. The assignment of the backbone ^1^H, ^15^N and ^13^C atoms was achieved by following the standard methodology. The 3D experiments HNCO, HNCA, HN(CO)CA, CBCA(CO)HN and CBCANH [79], recorded in the AV800 spectrometer, were manually assigned for Pub1p R12. An equivalent set of experiments, plus HN(CA)CO [79], was acquired for the Pub1p R3 construct, and the spectra were assigned by following a supervised automatic assignment protocol [80]. 3D HC(C)H-TOCSY and (H)CCH-TOCSY experiments [81] were recorded to assign the side chain resonances of Pub1p R3 construct. NMR spectra processing was done with NMRPipe [82] whereas spectral viewing and analysis were done with the program CcpNmr Analysis [83]. The completeness of the assignment for the Pub1p R3 construct extends to 95%, while only backbone ^13^C/^15^N resonances were assigned for the Pub1p R12 construct.

For Pub1p R3, a total of 1968 NOE-derived distance constraints were extracted from the 2D-NOESY [84] and ^15^N-edited-3D-NOESY [85] spectra, both with mixing times of 80 ms. Additional 129 dihedral angular constraints were extracted from the analysis of ^13^C (Cα ,C′ and Cβ) chemical shifts [86] and incorporated to the structure calculation. The structure calculation was performed by following an standard torsion angle dynamics protocol implemented in the program CYANA 2.1 [87]. Coordinates of 50 conformers were randomly generated and then subjected to the restrained simulated annealing protocol. The 20 lowest target function conformers with no distance violations larger than 0.2 Å and no angle violations larger than 5.0° were selected as representative of the structure of the protein (PDB code: 2LA4 and BMRB code: 17502). MOLMOL [88] and PyMOL v0.98 (DeLano Scientific LLC, Palo Alto, CA, USA) molecular graphics packages were used for the analysis and representation of the structures.

### Protein-RNA interactions analysis by NMR

All RNA probes were chemically synthesized and purchased from IDT (Integrated DNA technologies). All samples for NMR titration studies were prepared by simultaneously dialyzing against the same protein buffer (see above for other NMR experiments). Samples were prepared without D_2_O in a 4 mm NMR tube (typically 300 µl) and then inserting this into a 5 mm tube that contains D_2_O for referencing the lock signal. In the case of Pub1p R12, ^1^H-^15^N HSQC experiments were recorded at 1∶1 protein∶RNA ratio (100 µM concentration each) for the probes: U_14_, A_14_ and (UA)_7_. Assignment of the spectrum of the A_14_ complex was made by simple comparison with the free protein spectrum, whereas the Pub1p-U_14_ complex spectrum was assigned with the aid of triple resonance experiments (3D HNCA and CBCA(CO)HN) acquired with a concentrated 1∶1 sample (500 µM). Pub1p R3 RNA binding affinity and selectivity was investigated with eleven RNA probes: U_14_, U_12_, U_10_, A_14_, (UA)_7_, (UA)_6_, (CU)_6_, (AG)_4_, (AC)_4_, (GU)_4_ and C_8_. Experimental series were performed starting at the highest RNA∶protein ratio (typically 400 µM∶100 µM) and the following points prepared by dilution of this sample with a stock of ^15^N labelled protein at the same concentration and buffer composition, thus the protein concentration is kept fixed. Six to eight titration points were typically recorded for each RNA probe at protein∶RNA ratios ranging between 4∶1 and 0.03∶1. The weighted averaged ^1^H and ^15^N amide chemical shift changes were calculated according to the following formula:
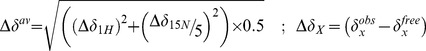
(1)


The Δδ^av^ values measured in experiments at constant protein concentration (P_0_) were plotted *versus* the total ligand concentration values (L_0_) and fitted to the following equation to obtain the maximum chemical shift change (Δδ_max_) and the dissociation constant (K_D_) [89]:
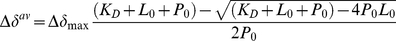
(2)


Because this equation applies only in the fast exchange limit [89], ^1^H-^15^N HSQC peaks experiencing line broadening during the titration were excluded from the K_D_ calculation. The remaining peaks showing Δδ^av^>0.1 ppm between the free and the RNA-bound protein at the highest RNA/protein ratio were fit globally (K_D_ global parameter; Δδ_max_ local parameter for each curve) to equation (2) by a non-linear least squares method with the software package GraphPad Prism version 5.00 for Mac (GraphPad Software, San Diego California USA, www.graphpad.com).

### Protein-protein interactions analysis by NMR

Samples for the protein constructs Pub1p R12, Pub1p R3 and Tif4631p (1–402) were dialyzed simultaneously against 25 mM potassium phosphate buffer (pH 7.0, 25 mM NaCl, 0.1 mM DTT) for 24 hours at 4°C twice, to guarantee no differences in buffer composition. Binding was monitored on the ^1^H-^15^N HSQC spectra of Pub1p R12 and Pub1p R3 upon titration with different amounts of unlabeled Tif4631p (1–402). Pub1p constructs were prepared at 100 µM concentration and Tif4631p (1–402) was added at different ratios: 1∶2 for the Pub1p R12-Tif4631p (1–402) complex and 1∶1.9, 1∶0.8 and 1∶0.4 for the Pub1p R3-Tif4631p (1–402) one. The chemical shift perturbations were calculated as weighted average of ^1^H and ^15^N amide chemical shift changes (equation (1)). A complementary NMR titration experiment was done by monitoring changes in the ^1^H-^15^N HSQC spectrum of 100 µM ^15^N-labelled Tif4631p (1–402) upon addition of 120 µM unlabelled Pub1p R3. All the NMR experiments were recorded at 25°C in a Bruker AV800 spectrometer with a cryoprobe unit.

### *In vitro* protein-protein cross-linking experiments

Pub1p-Tif4631p interaction was probed by glutaraldehyde cross-linking using a variation of the vapor diffusion method [67]. Sample volumes of 200 µL were prepared by mixing different amounts of Pub1p and Tif4631p constructs in 25 mM potassium phosphate, 150 mM NaCl and 0.1 mM DTT buffer (pH 8.0) on ice. Pub1p proteins (Pub1p R3, Pub1p R12 and Pub1p R3 Phe364Ala/Phe366Ala mutant) were kept at 10 µM final concentration in all the experiments, Tif4631p (1–402) at 1 µM and Tif4631p (1–184) at 4 µM. The concentration of the Tif4631p proteins was experimentally optimized in an attempt to compensate the larger number of potentially reactive side chains in these proteins (41 and 13 Lys respectively) in comparison with Pub1p R3 (4 Lys) and Pub1p R12 (12 Lys) constructs. The different protein mixtures were loaded in the buffer reservoirs of a 24-well sitting-drop plate (Hampton Reserch), the upper reservoir was filled with 20 µL of 0.5 M aqueous glutaraldehyde solution, the plate sealed with film and incubated 16 h at 4°C allowing a smooth diffusion of the cross-linker. Upon reaction completion, the glutaraldehyde drops were carefully removed and 10 µL of 2 M Tris-HCl (pH 8.0) were added to the reaction mixture to quench the traces of reactant in the protein mixture. Sample (10 µl) components were separated by PAGE-SDS electrophoresis (15% gel) and revealed by silver stain methods. Reaction mixtures were further characterized by MALDI-TOF MS.

### Circular dichroism measurements

All CD experiments were recorded in a Jasco J-810 spectropolarimeter equipped with a Peltier temperature control unit. Thermal denaturation experiments were carried out in a 25 mM saline potassium phosphate buffer (pH 7.5–8.0) at protein concentrations of approximately 40 µM. Cell path lengths of 0.1 cm (Pub1p R3 wild-type) and 0.5 cm (Pub1p R3 Trp410Phe) were used. Ellipticity was recorded at 220 nm at a temperature range of 20–95°C for wild-type and 20–80°C for mutant protein and at heating rates of 60°C/hour and 30°C/hour respectively.

## Supporting Information

Figure S1 **Protein solubility and stability of Trp410 mutants.** (**A**). Summary of the protein expression test performed on various Trp410 single mutants in *E. coli* BL21 (DE3) strains. All proteins were expressed as thioredoxin fusions and gels show the levels of total and soluble fractions obtained after induction of protein expression at two temperatures. (**B**) Melting curves of wild-type (left) and W410F mutant (right) proteins monitored by circular dichroism (arbitrary ellipticity units).
(PDF)

Figure S2 **Pub1p R3 Phe364Ala/Phe366Ala mutant maintains the 3D structure of the wild-type protein.** The comparison of the aromatic region of the 2D TOCSY spectra of the two proteins shows most of the Tyr and Phe side chains at very similar positions, indicative of their structurally similar environments. The residue Phe 361, spatially close to the mutations, suffers the largest perturbation in this region.
(PDF)

Figure S3 **Control cross-linking reactions of individual protein constructs used in this study.** (**A**) Silver-stained 15% PAGE gel comparing glutaraldehyde treated and untreated Pub1p constructs. Conditions are the same as in Figure 7 and in Materials and Methods. (**B**) Same comparison for the three Tif4631p constructs. The approximate position of the Tif4631p (1–82) band was indicated with an asterisk. This constructs was not stained with silver and is only visible (by negative staining) in heavily stained gels (data not shown).
(PDF)

Figure S4 **Several examples of RRM mediated protein-protein recognition modes.** (**A**) Tif4631p recognition interface of Pub1p (shaded in green), (**B**) complex between FBP (green) and FBP-interacting repressor (FIR) (in grey) and (**C**) complex between SPF45 UHM domain (grey) and a SF3b-155 peptide (green).
(PDF)
