# Supplementary figures and images for: Pub1p C-Terminal RRM Domain Interacts with Tif4631p through a Conserved Region Neighbouring the Pab1p Binding Site
Source: PLoS One. 2011 Sep 8;6(9):e24481. doi: 10.1371/journal.pone.0024481 (PMC3169606; doi:10.1371/journal.pone.0024481)

Supplementary figure 1

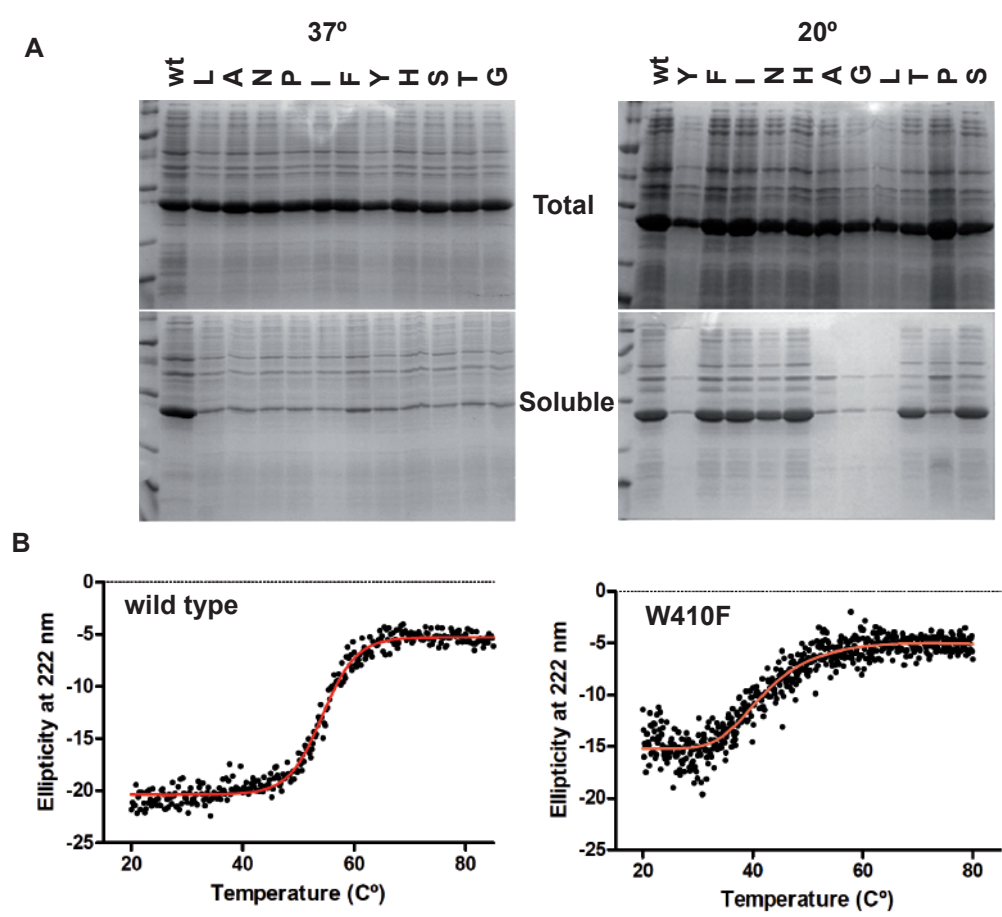

Supplement: Figure S1 — Protein solubility and stability of Trp410 mutants. (A). Summary of the protein expression test performed on various Trp410 single mutants in E. coli BL21 (DE3) strains. All proteins were expressed as thioredoxin fusions and gels show the levels of total and soluble fractions obtained after induction of protein expression at two temperatures. (B) Melting curves of wild-type (left) and W410F mutant (right) proteins monitored by circular dichroism (arbitrary ellipticity units). (PDF) [file pone.0024481.s001.pdf]

**A**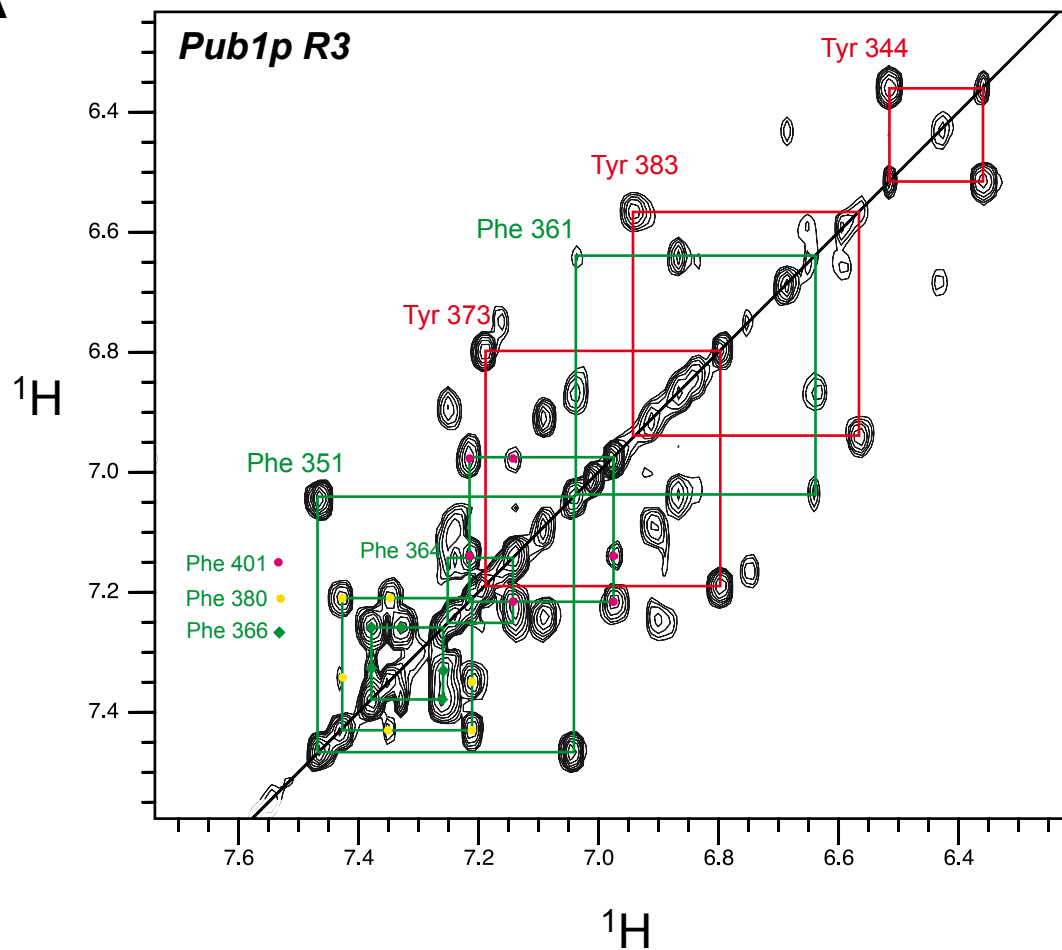**B**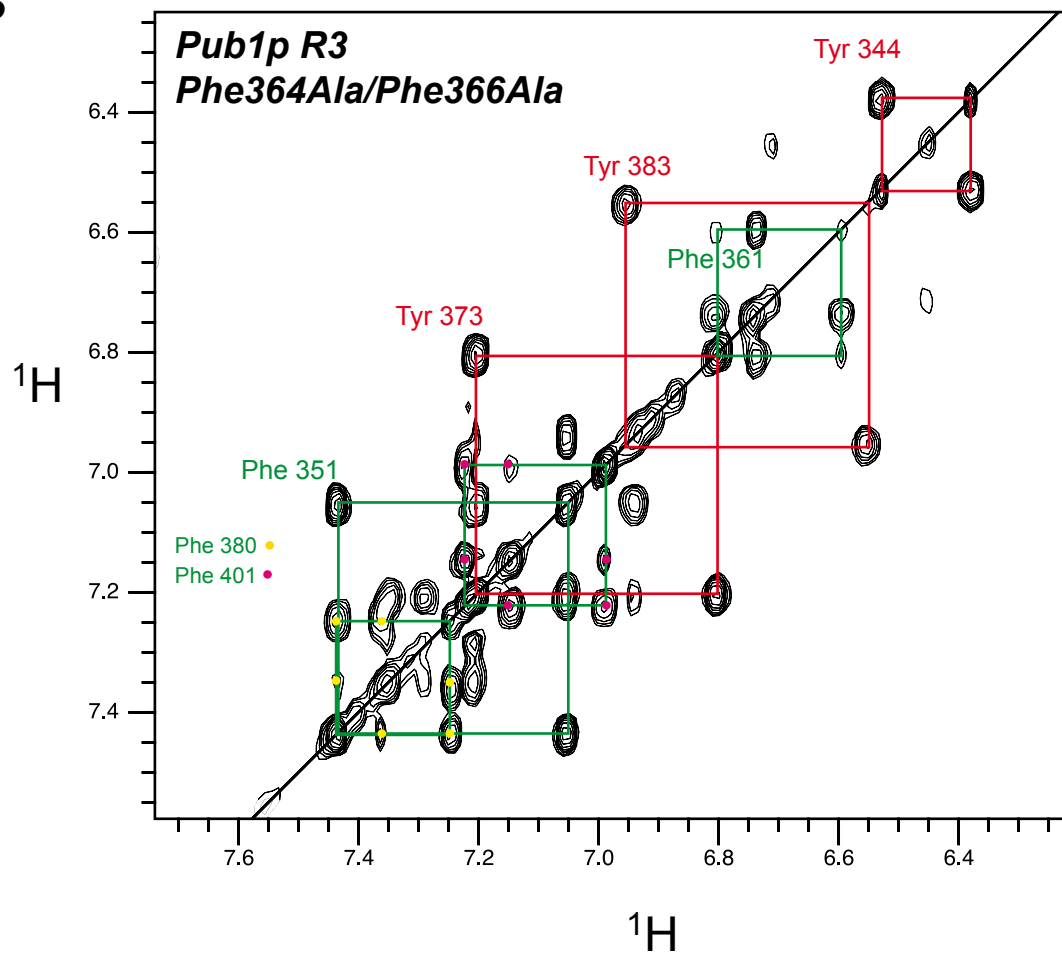

Supplement: Figure S2 — Pub1p R3 Phe364Ala/Phe366Ala mutant maintains the 3D structure of the wild-type protein. The comparison of the aromatic region of the 2D TOCSY spectra of the two proteins shows most of the Tyr and Phe side chains at very similar positions, indicative of their structurally similar environments. The residue Phe 361, spatially close to the mutations, suffers the largest perturbation in this region. (PDF) [file pone.0024481.s002.pdf]

**A**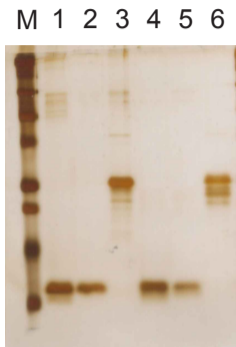

|                     |   |   |   |   |   |   |
|---------------------|---|---|---|---|---|---|
| Pub1p R3            | + | - | - | + | - | - |
| Pub1p R3 F364,366A. | - | + | - | - | + | - |
| Pub1p R12           | - | - | + | - | - | + |
| Glutaraldehyde      | - | - | - | + | + | + |

**B**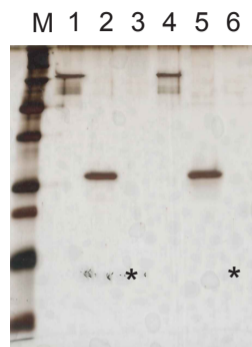

|                  |   |   |   |   |   |   |
|------------------|---|---|---|---|---|---|
| Tif4631p (1-402) | + | - | - | + | - | - |
| Tif4631p (1-184) | - | + | - | - | + | - |
| Tif4631p (1-82)  | - | - | + | - | - | + |
| Glutaraldehyde   | - | - | - | + | + | + |

**Figure S3**

Supplement: Figure S3 — Control cross-linking reactions of individual protein constructs used in this study. (A) Silver-stained 15% PAGE gel comparing glutaraldehyde treated and untreated Pub1p constructs. Conditions are the same as in Figure 7 and in Materials and Methods. (B) Same comparison for the three Tif4631p constructs. The approximate position of the Tif4631p (1–82) band was indicated with an asterisk. This constructs was not stained with silver and is only visible (by negative staining) in heavily stained gels (data not shown). (PDF) [file pone.0024481.s003.pdf]

Supplementary figure 2

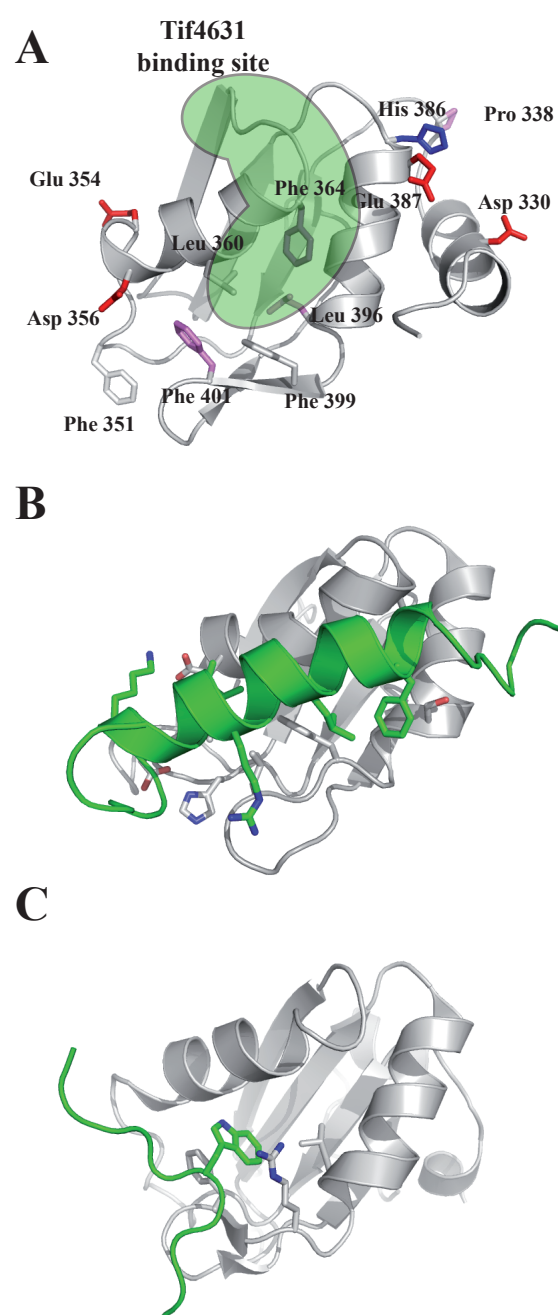

Supplement: Figure S4 — Several examples of RRM mediated protein-protein recognition modes. (A) Tif4631p recognition interface of Pub1p (shaded in green), (B) complex between FBP (green) and FBP-interacting repressor (FIR) (in grey) and (C) complex between SPF45 UHM domain (grey) and a SF3b-155 peptide (green). (PDF) [file pone.0024481.s004.pdf]
